# Hydrogen-mediated magnetic domain formation and domain wall motion in Co_30_Pd_70_ alloy films

**DOI:** 10.1038/s41598-018-25114-3

**Published:** 2018-04-27

**Authors:** Po-Chun Chang, Chak-Ming Liu, Chuan-Che Hsu, Wen-Chin Lin

**Affiliations:** Department of Physics, National Taiwan Normal University, Taipei, 11677 Taiwan

## Abstract

In this study, the microscopic origin of the hydrogen effect on magnetic materials was explored through the characterization of time-dependent magnetic domain evolution. We prepared 25-nm Co_30_Pd_70_ alloy films with canted magnetic moment on SiO_2_/Si(001) substrates. From macroscopic Kerr hysteresis loops, considerable hydrogen-induced reduction of magnetic coercivity by a factor of 1/5 in a longitudinal direction and enhancement of magnetic remanence to saturation ratio from 60% to 100% were observed. The magnetic reversal behavior of the Co_30_Pd_70_ alloy films gradually transformed from nucleation- to domain-wall-motion dominance when H_2_ pressure was increased from a vacuum of 1 × 10^−5^ mbar to 0.8 bar. Domain size also increased considerably with H_2_ pressure. When H_2_ pressure was above 0.4 bar, the domain wall (DW) motion was clear to observe and the DW velocity was approximately 10^−6^–10^−5^ m/s. Greater hydrogen content in the Co_30_Pd_70_ alloy films promoted DW motion that was closer to the behavior of a thermally activated model. The hydrogen effects on magnetism were observed to be reversible and could have valuable future application in spintronic devices for hydrogen sensing.

## Introduction

Owing to the strength of the interaction between Pd and hydrogen atoms, Pd has been used as an efficient catalyst for the dissociation of hydrogen molecules into individual hydrogen atoms^1–4^. Gaseous H_2_ molecules adsorbed onto the metallic Pd surface and then dissociated through the formation of Pd-H hydride^1^. The small radius of a single hydrogen atom (approximately 1.1 Å) is comparable to the interstitial space between Pd atoms. Thus, at room temperature, hydrogen atoms can easily diffuse in the Pd lattice; hydrogenation is extended into the volume of Pd materials.

Pd-related alloys have also been studied because of their potential in the fields of hydrogen sensing and hydrogen storage. When Pd is combined with other elements, further hydrogen-induced functionality and applicable properties may be found^5,6^. For example, hydrogen changes the magnetic interlayer interaction of Fe/Nb and Fe/V multilayers from antiferromagnetic to ferromagnetic coupling^7–9^. In magnetic Pd alloys, such as Fe_*x*_Pd_1−*x*_ and Co_*x*_Pd_1−*x*_ alloys, considerable attention has been paid to the reversible hydrogen effect on magnetic properties^10–15^. Lueng *et al*. reported that nanopatterned Pd/Co films were candidates for future hydrogen gas sensing devices based upon their hydrogen-absorption-modified ferromagnetic resonance^16^. Their qualitative analysis demonstrated that the magnetoelastic contribution to hydrogen-induced change in perpendicular magnetic anisotropy (PMA) was negligible^17^. Munbodh *et al*. studied CoPd multilayers and found that H_2_ desorption reduced both the PMA and total magnetization of samples^18,19^. Liang *et al*. studied the magnetic and electronic properties of a giant magnetoresistance device [Co_40_Pd_60_/Cu]_10_/Fe for use in the detection of H_2_. The applicability of a spintronic device as a low-pressure H_2_ detector was also demonstrated based on its sharp and reproducible H_2_-dependent magnetic and electrical properties; specifically, the effect of hydrogenation in a CoPd alloy can be viewed as a spin-dependent charge transfer^20^. Some other studies have also demonstrated that hydrogen-induced modulation of the spin polarization of Co leads to various changes in global magnetism, magnetic susceptibility, magnetic anisotropy, and transition temperature^13,21,22^. Gerber *et al*. presented the concept of magnetic gas detection by using the extraordinary Hall effect. The Hall effect sensitivity of the optimized samples exceeded 240% per 10^4^ parts per million at hydrogen concentrations below 0.5% in a hydrogen/nitrogen atmosphere, which is more than two orders of magnitude higher than the sensitivity of the conductance detection^5^.

In our previous studies, hydrogenation did not trigger an observable change in the magnetic behavior in simple bilayer (Pd/Fe, Pd/Co, or Pd/Ni) or as-grown trilayer (Pd/Co/Pd) magnetic structures^6,23,24^. Only modulation of the magneto-optical effect was observed in these systems due to the hydrogen-induced change in optical properties. Moreover, a significant change in magnetic properties in Pd-rich alloys was found when exposed to H_2_ gas. For example, hydrogen induced reversible spin reorientation transition in Co_50_Pd_50_ alloy thin films^22^. Exposure to H_2_ gas also transformed the short-range coupled and disordered magnetic state of the Co_14_Pd_86_ film to a long-range-ordered ferromagnetic state and induced an appreciable decrease in the magnetic moment^14,15^.

All the aforementioned research reported the effect of hydrogen on macroscopic magnetic properties, such as collective magnetic hysteresis loops, averaged magnetic moment, and ferromagnetic resonance. These results demonstrated the potential of using spintronic materials in hydrogen-related technologies. However, microscopic characterization of the effects of hydrogen on magnetism is still challenging. In the present study, a magneto-optical Kerr effect (MOKE) microscope with a vacuum-sealed sample holder was used for the characterization of magnetic domain evolution under various magnetic fields and hydrogen gas pressure. Hydrogen absorption in a Pd-rich alloy thin film can change the mechanism of magnetic domain reversal and mediate the domain wall (DW) motion. The experimental results fitted with theoretical models and the transition of domain reversal behavior are discussed in this paper.

## Results

### Hydrogen effect on magnetic hysteresis loops

**Figure 1:**
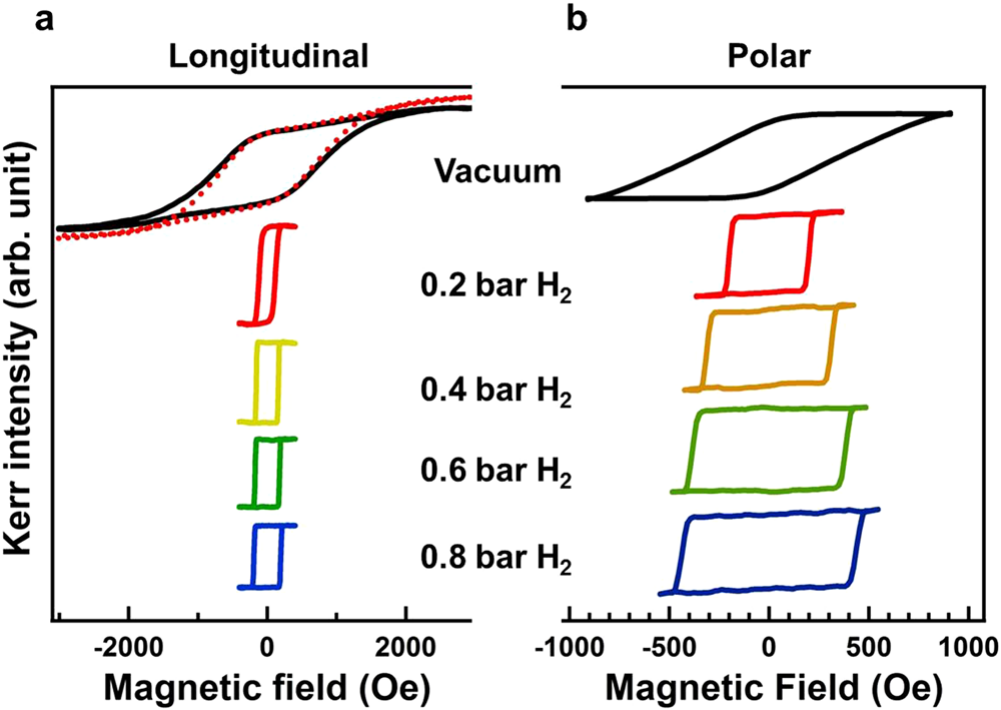
(**a**) Longitudinal and (**b**) polar MOKE hysteresis loops measured under a vacuum and various H_2_ pressures (0.2–0.8 bar). The dashed red curve in (**a**) represented the data measured after the recovery to a vacuum and was nearly the same as the pristine hysteresis loop of the initial vacuum condition (solid black curve).

**Figure 2:**
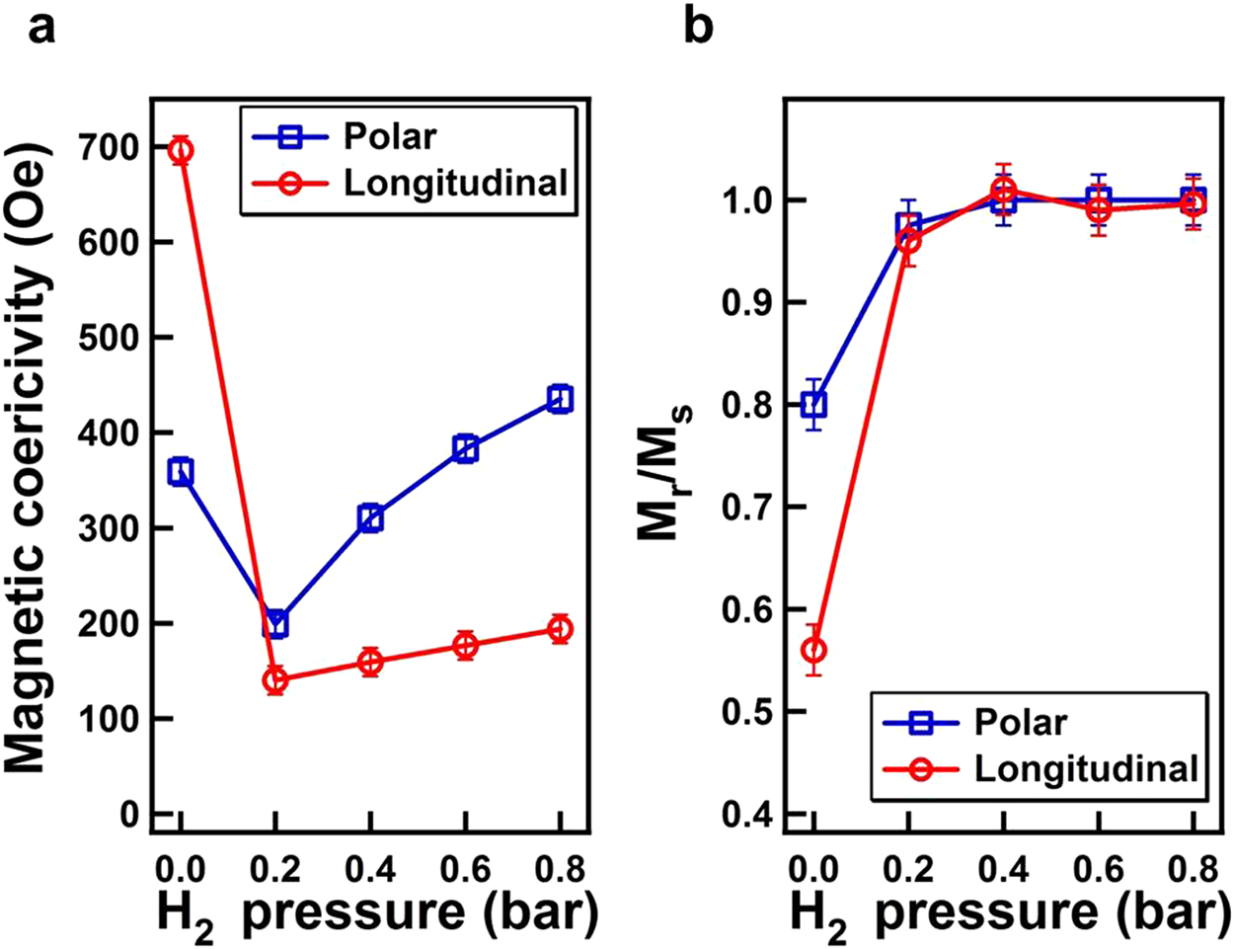
(**a**) Longitudinal and (**b**) polar magnetic coercivity (H_*c*_) deduced from the hysteresis loops in Fig. 1 plotted as a function of H_2_ pressure.

Figure 1(a) and (b) exhibit the longitudinal and polar magnetic hysteresis loops of a 25 nm Co_30_Pd_70_ alloy film measured in a vacuum, under 0.2–0.8 bar H_2_ pressure, and then again in the vacuum. Figure 2 summarizes the magnetic coercivity (H_*c*_) values of the hysteresis loops as a function of hydrogen pressure. In the initial vacuum condition, the longitudinal hysteresis loop was tilted, and the magnetic coercivity reached 700 Oe. In the polar direction, the maximum magnetic field was not sufficient to saturate the magnetization, and the H_*c*_ should have been more than the measured value of 450 Oe. When the Co_30_Pd_70_ alloy film was exposed to an environment filled with 0.2 bar H_2_ gas, the magnetic hysteresis loops became square, in which the remanence magnetization sustained the same value as the saturation value and magnetization reversal switched sharply as the magnetic field was close to the H_*c*_. Compared with the initial vacuum condition, exposure to 0.2 bar H_2_ considerably reduced the longitudinal H_*c*_ from 700 Oe to approximately 140 Oe. The marked change in the H_*c*_ and the improved squareness implied a transition of magnetization reversal mechanism, triggered by the hydrogen content in the Co_30_Pd_70_ alloy film. With the increase in H_2_ pressure from 0.2 to 0.8 bar, the magnetic hysteresis loops remained in a square-like shape and the H_*c*_ monotonically increased from 140 Oe to 194 Oe and from 200 Oe to 435 Oe for the longitudinal and polar geometry, respectively. The coexistence of magnetic hysteresis loops in both directions and their comparable H_*c*_, indicated that the magnetization was stabilized in a direction canted from the surface normal. Note that the dashed red hysteresis loop in Fig. 1(a) represented the data measured after the recovery to a vacuum and was nearly the same as the pristine hysteresis loop of the initial vacuum condition (solid black curve). This demonstrated the reversibility of hydrogen effect on the magnetic behavior.

Figures 1 and 2 indicate that the hydrogen effects on a 25-nm Co_30_Pd_70_ alloy film included the drastic reduction of H_*c*_ and the transformation of remanence to nearly 100% of the saturation upon exposure to 0.2 bar H_2_; when the H_2_ pressure was increased from 0.2 to 0.8 bar, the H_*c*_ monotonically slightly increased. Similar results were found in research on approximately 15 nm Co_14_Pd_86_ alloy films on Al_2_O_3_(0001); exposure to 40 mbar H_2_ induced transformation to 100% squareness of the hysteresis loops with a relatively small H_*c*_, and subsequent increases in H_2_ pressure monotonically increased the H_*c*_ toward larger values. The consistent hydrogen effects of 100% remanence to saturation ratio in the present Co_30_Pd_70_/SiO_2_/Si(001) and the previously reported Co_14_Pd_86_/Al_2_O_3_(0001) indicated that the absorbed hydrogen content in the CoPd alloy promoted long-range magnetic coupling. In the current study, aside from hydrogenation, thermal annealing of a Co_30_Pd_70_ film at 700 K also induced considerable H_*c*_ reduction and 100% squareness of the hysteresis loop. Thermal annealing of the magnetic thin film usually improves the crystalline ordering or the intergrain coupling and thus changes the magnetic behavior. In contrast to the irreversible annealing effect, H_2_ exposure provided a reversible method to mediate and strengthen magnetic coupling in magnetic materials.

### Longitudinal magnetic domain reversal

**Figure 3:**
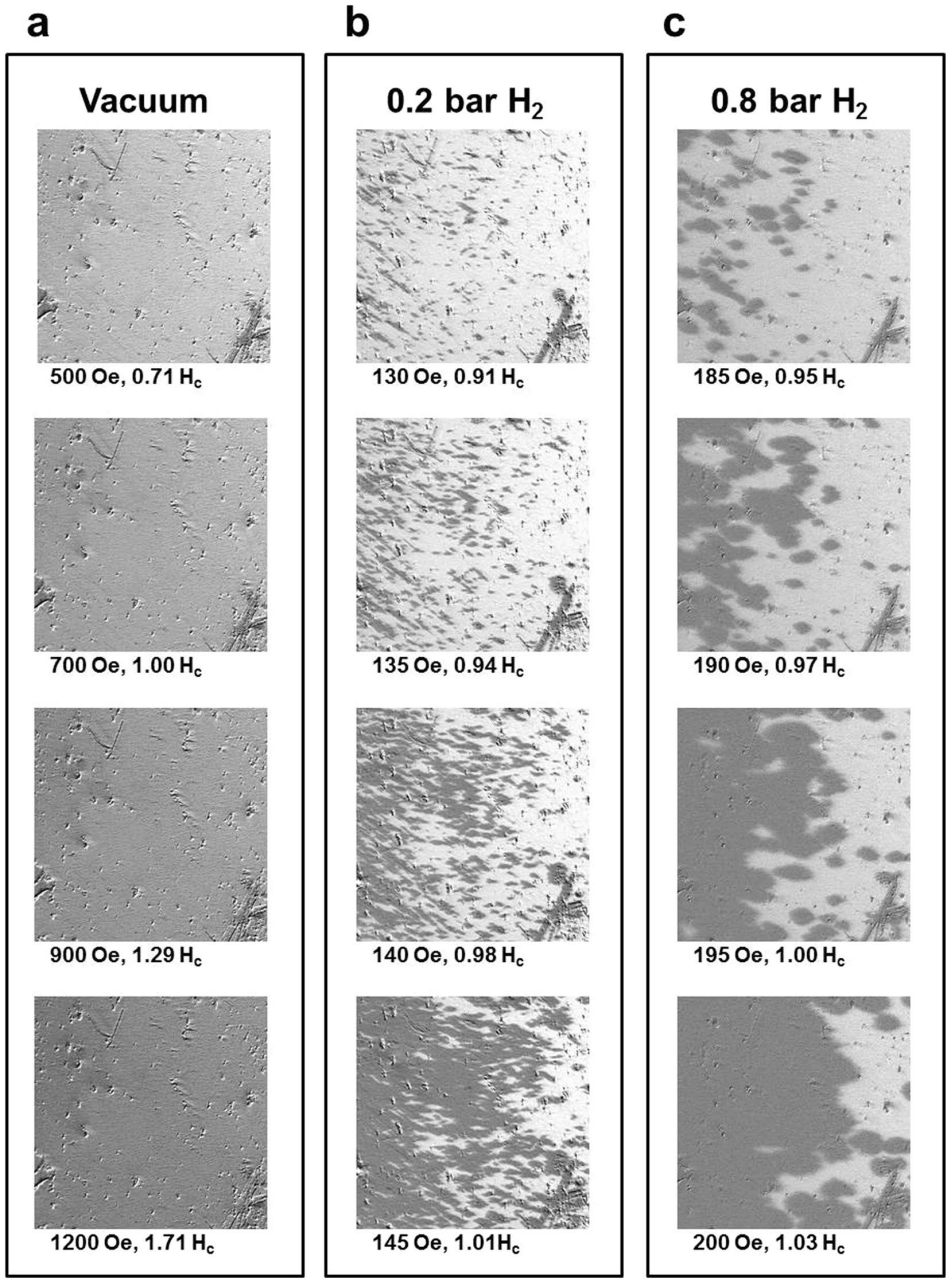
Magnetic-field-dependent longitudinal Kerr images taken under (**a**) a vacuum, (**b**) 0.2 bar H_2_ and (**c**) 0.8 bar H_2_. All Kerr images were taken over the same area, 0.2 s after the application of a magnetic field. The image size was 450 × 450 *μ*m^2^.

Figures 1 and 2 exhibit the considerable hydrogen effect on the magnetic hysteresis loop of a 25-nm Co_30_Pd_70_ alloy film. Because the hysteresis loops were averaged from the mm-scale collective behavior, the characterization of microscope magnetic behavior can help to clarify the hydrogen effect mechanism. Figure 3 exhibits three magnetic-field-dependent Kerr images of a 25-nm Co_30_Pd_70_ alloy film measured in a longitudinal direction under (a) a vacuum, (b) a H_2_ pressure of 0.2, and (c) 0.8 bar, respectively. The images were taken over the same 450 × 450 *μm*^2^ area. The defect in the right-lower corner of the images confirmed that the focused area remained the same in different series of measurement. Before measurement, the sample was fully saturated with a larger positive magnetic field. Subsequently, the magnetic field was gradually decreased to zero and then negatively increased to observe the magnetic domain structure during magnetization reversal. As the applied magnetic field was close to the H_*c*_ value, the magnetic domain was expected to be apparent from the Kerr images. The series of Kerr images exhibit three magnetization reversal mechanisms. The Co_30_Pd_70_ alloy film under a vacuum, as shown in Fig. 3(a), presented no observable domain structure; the gray scale gradually and continuously became darker with the negative increase in the magnetic field. As long as the Co_30_Pd_70_ alloy film was under a vacuum, no matter in the as deposited condition or after hydrogen cycles, there was always no observable domain structure in MOKE measurement. The absence of the magnetic domain structure was due to the magnetization reversal being dominated by small nucleations of reverse magnetization with limited, unobservable DW motion (i.e., domain extension). The reversal nucleations were randomly distributed on the thin film, and their size was of a submicrometer scale below the resolution of the MOKE microscope to ensure that only the magnetic field-driven gray scale change in the Kerr images were observed without any noticeable contrasted magnetic domain structure.

For the sample exposed to 0.2 bar H_2_, as shown in Fig. 3(b), small needle-like domain structures were seen with a magnetic field of 0.91 H_*c*_. The number and size of dark domains gradually increased when the magnetic field reached 0.98 H_*c*_. With the field at 1.01 H_*c*_, the discontinuous domains expanded and merged with the nearest ones, and the reversal domain dominated the image area. In contrast to the absence of magnetic domain structure under a vacuum, 0.2 bar H_2_ induced visible magnetic reversal nucleations; the size ranged from a few *μ*m to tens of *μ*m. A small magnetic field was sufficient to create magnetic reversal nucleations, and crucially, the presence of hydrogen content also advanced the DW motion, leading to the expansion of reversal nucleations and subsequently the large magnetic domains.

For the sample exposed to 0.8 bar H_2_, as shown in Fig. 3(c), with a magnetic field of 0.91 H_*c*_, the appearance of the domain was not noticeably more needlelike; the domain became diamond shaped and was larger than that under 0.2 bar H_2_. When the magnetic field was increased to 0.97–1.0 H_*c*_, instead of the appearance of new small domains, the pristine domain rapidly expanded and merged. Compared with the high magnetic reversal nucleation density and small size with needle-like shape under 0.2 bar H_2_, exposure to 0.8 bar H_2_ promoted larger nucleation size; however, the nucleation density was low. This indicated that the magnetic DW motion became dominant in the magnetization reversal process. The series of Kerr images in Fig. 3(a–c) demonstrate that hydrogen induced the transition of domain reversal mechanism from nucleation dominance to DW-motion dominance.

**Figure 4:**
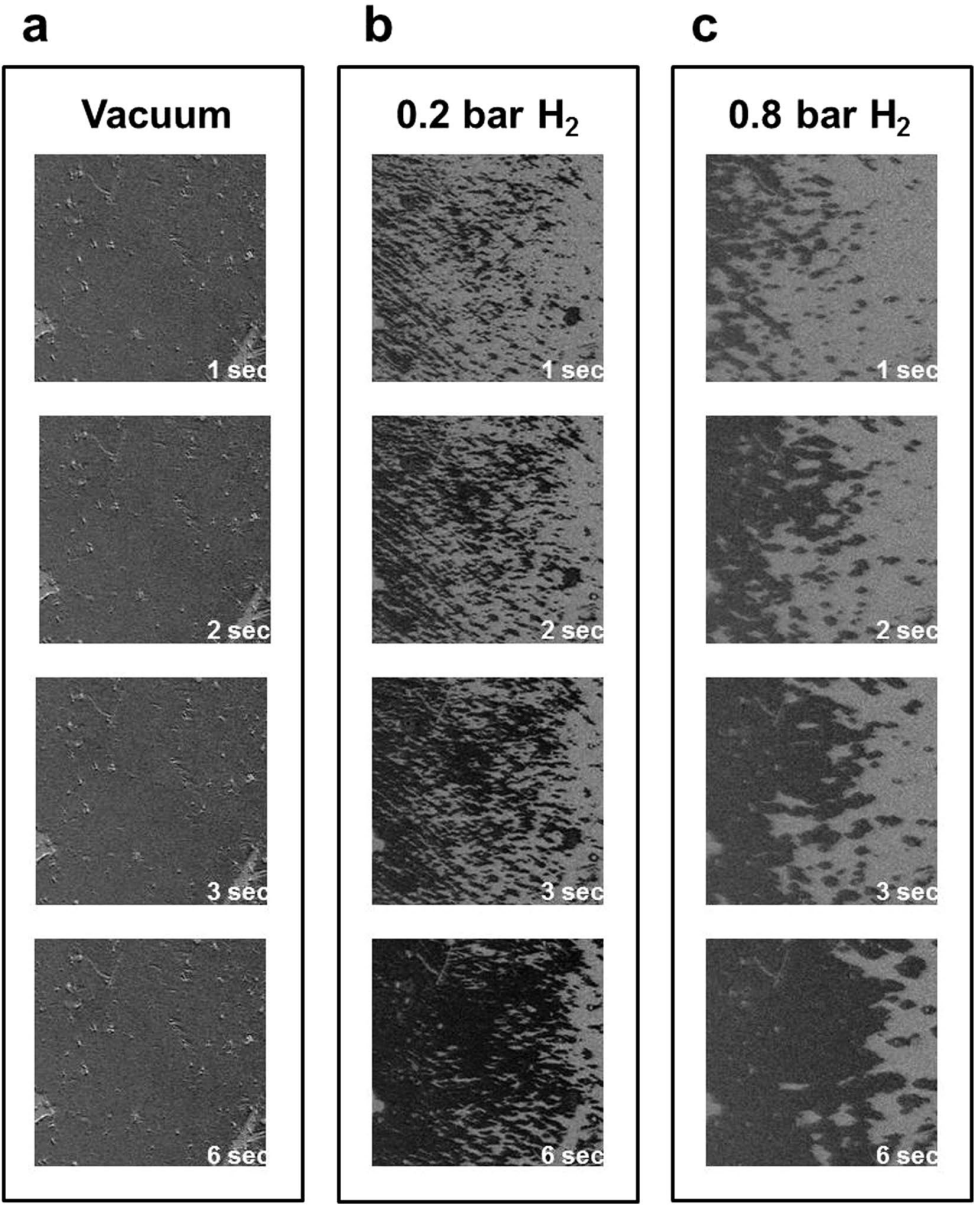
Time-dependent longitudinal Kerr images taken under. (**a**) a vacuum, (**b**) 0.2 bar H_2_ and (**c**) 0.8 bar H_2_. The three series of Kerr images were taken over the same area, 1, 2, 3, and 6 s after the application of the magnetic field, which was 0.9 times the magnetic coercivity field in each condition. The image size was 450 × 450 *μ*m^2^.

To conduct advanced analysis of the hydrogen-mediated magnetic DW motion, the time-dependent magnetic domain reversal processes were monitored with the various applied magnetic fields and under different H_2_ gas pressures. Figure 4(a) shows the Kerr images taken under a vacuum with an applied magnetic field of 0.9 H_*c*_. No observable magnetic domain contrast presented at 1–6 s after the application of the negative magnetic field. Moreover, no observable magnetic domain was observed under a vacuum, regardless of how large a magnetic field was applied or the length of time that passed after the field was applied. In Fig. 4(b), under exposure to 0.2 bar H_2_, 1 second after the application of a magnetic field of 0.9 H_*c*_, numerous needle-shape reversal nucleations appeared. Over the following 2–6 s, the nucleation number (density) was nearly unchanged, and the dark domain (reversal domain) gradually extended by DW motion. In Fig. 4(c), under exposure to 0.8 bar H_2_, 1 s after the application of a magnetic field of 0.9 H_*c*_, reversal nucleations spread over the film. Compared with the condition of 0.2 bar H_2_ in Fig. 4(b), exposure to 0.8 bar H_2_ led to less nucleation density, but a relatively large domain size. DW motion dominated the magnetization reversal under 0.8 bar H_2_.

**Figure 5:**
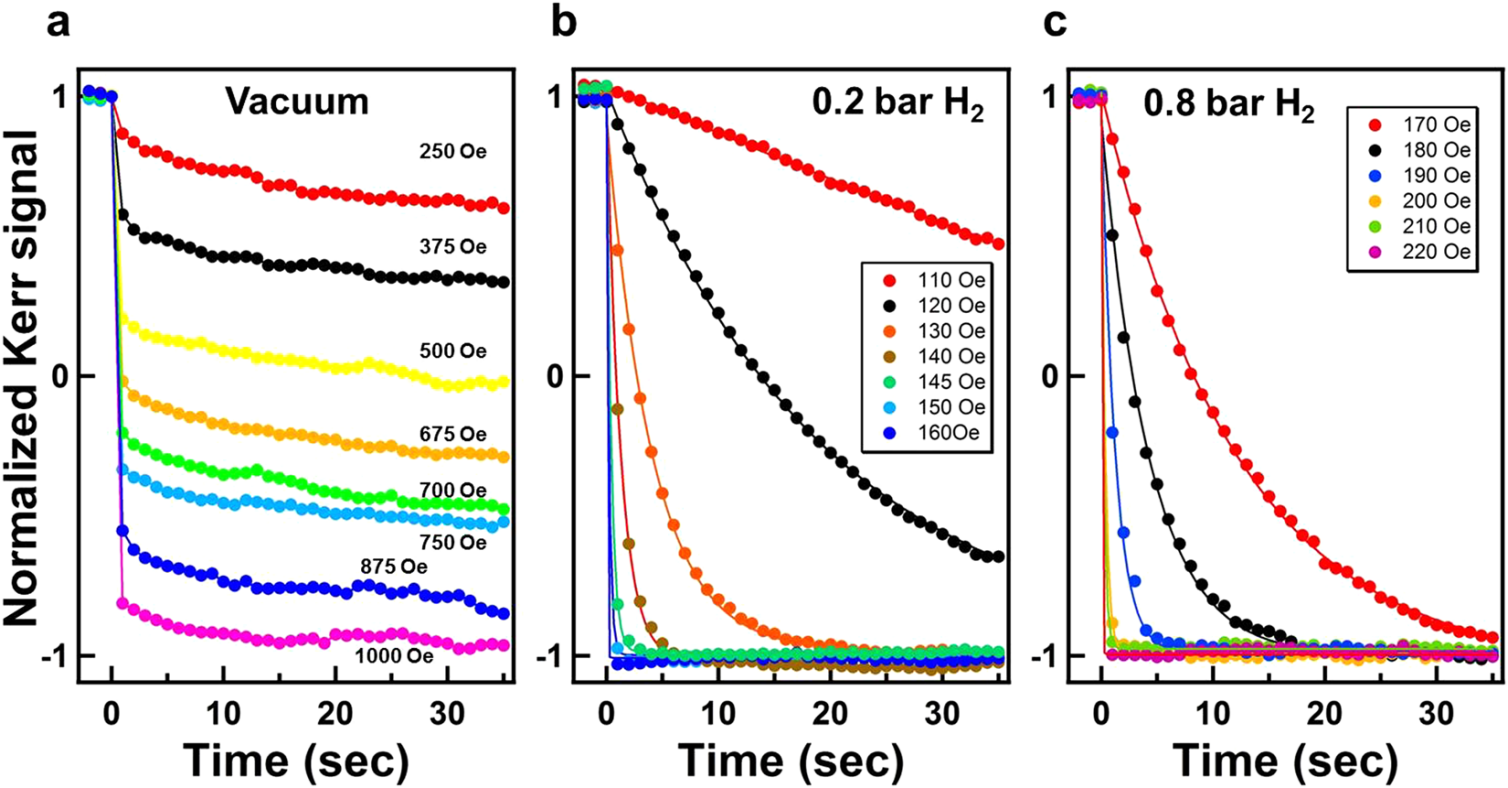
Time-dependent magnetization reversal curves recorded under (**a**) a vacuum (**b**) 0.2 bar H_2_ and (**c**) 0.8 bar H_2_ after the application of various reverse magnetic fields. The normalized Kerr intensity was obtained from the intensity integration of the Kerr images in Fig. 4.

Quantitative analysis of the time-dependent domain evolution in Fig. 4 can be performed by integrating the intensity of the Kerr images to obtain the average domain reversal rate. The average magnetization was recorded as a function of time after the reversal magnetic field was applied. Before the magnetization reversal, the sample was fully saturated with a large positive field (3000 Oe), subsequently retained at the remanence state (0 Oe), and then a negative magnetic field was applied to trigger the domain evolution. Figure 5 exhibits the time-dependent magnetization reversal curves with the application of various magnetic fields under (a) a vacuum, (b) 0.2 bar H_2_ and (c) 0.8 bar H_2_. Due to the 100% magnetization at the remanence state, the reversal curves started from a fully magnetized +*M*_*s*_ state and then gradually switched to inverse magnetization (i.e., toward −*M*_*s*_ state). As previously proposed by Bruno *et al*., time-dependent magnetization reversal can be described by a kinetic model as follows^25,26^:

1 $$M(t)=2{M}_{s}\cdot \exp \,(\,-\,t/\tau )-{M}_{s}$$

where *M*_*s*_ denotes the average saturation magnetic moment and *τ* is the time constant. In Fig. 5(b) and (c), the solid curves exhibit the fitting results of the time-dependent magnetization reversal data using Eq. (1). When the larger reversal magnetic field was applied, the magnetization reversal completed faster and the smaller *τ* was obtained in the fitting. When only a free parameter *τ* is used, Eq. (1) can still successfully fit all the series of data in Fig. 5(b) and (c). Such a finding indicates that the proposed model, which is discussed later in this section, accurately described the magnetization reversal behavior under 0.2–0.8 bar H_2_. However, for the magnetization reversal curve measured in a vacuum, as exhibited in Fig. 5(a), the magnetization reduced drastically immediately after the application of the reversal field, then slowly decreased with a nearly constant slope, and was almost invariant for the variant magnetic field. The magnetization reversal curves in Fig. 5(a) exhibited entirely different behavior from that described by Eq. (1). Such a discrepancy is in accordance with Figs 3(a) and 4(a), where the magnetization reversal under a vacuum was dominated by submicrometer scale nucleations without observable DW motion. Nucleations reversed the magnetization immediately after (<1 s) the magnetic field was applied; this accounts for the drastic jumps in the first second of data in Fig. 5(a). In contrast to the nucleation dominance under a vacuum, when the Co_30_Pd_70_ alloy films were exposed to 0.2–0.8 bar H_2_ gas, DW motion was observed and dominated the magnetization reversal. Thus, Eq. (1) can successfully fit the data curves in Fig. 5(b) and (c).

The time constant *τ* depends on the activation energy E_*A*_ for the magnetic flipping of each microscopic element compared with the thermal energy *k*_*B*_ ⋅ *T*^25,26^.

2 $$\tau ={\tau }_{0}\cdot \exp \,(\,-\,{E}_{A}/{k}_{B}T)$$

The activation energy E_*A*_ varies linearly with the magnetic field *H* applied in the magnetization reversal process, as shown in Eq. (3)^25,26^.

3 $${E}_{A}=V\cdot {M}_{s}\cdot (H+{H}_{p})$$

where *V* is the Barkhausen volume, the magnetization of which is reversed within a single activation event. H_*p*_ is the field required for magnetization reversal in the absence of any activation processes. From the combination of Eqs (2) and (3), we can obtain the following relation between the time constant *τ* and the applied magnetic field *H*. *ln*(*τ*) is linearly correlated with *H*^25,26^.

4 $$ln(\tau )=-\,(\frac{V\cdot {M}_{s}}{{k}_{B}\cdot T})\cdot H+ln({\tau }_{0})-\,\frac{V\cdot {M}_{s}\cdot {H}_{p}}{{k}_{B}\cdot T}$$

**Figure 6:**
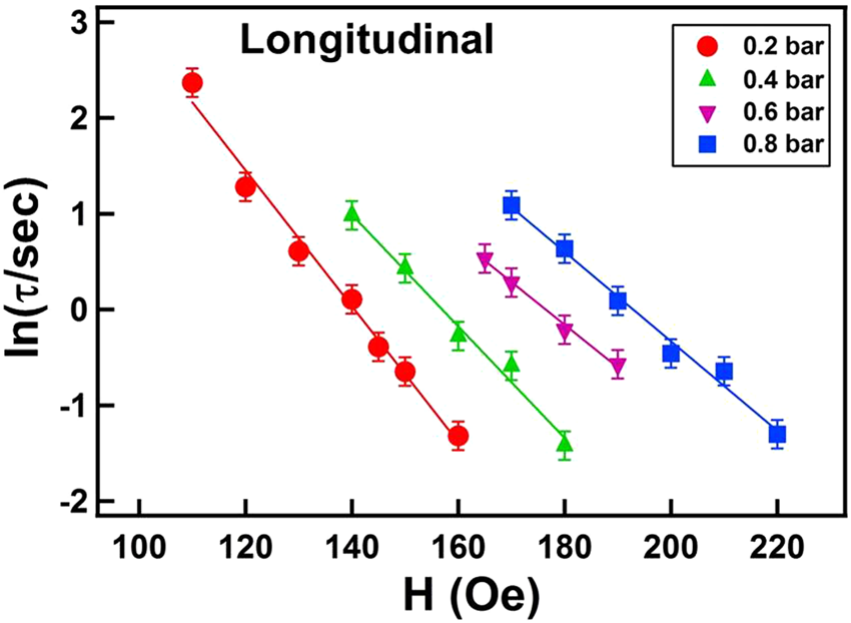
Taken from the curve fitting in Fig. 5 by Eq. (1), a series of time constant *τ* under various magnetic fields and H_2_ pressures. According to Eq. (4), *ln*(*τ*) was plotted as a function of the applied magnetic field *H*. The dots represent the experimental data and the solid lines represent the linear fitting results.

From the curve fitting in Fig. 5(b) and (c), a series of *τ* values under the various magnetic fields and H_2_ pressures were obtained. Accordingly *ln*(*τ*) was plotted as a function of the applied magnetic field H in Fig. 6. The four series of data behaved as expected from the linear function of Eq. (4). From the linear proportional parameter −(*V* ⋅ *M*_*s*_/*k*_*B*_ ⋅ *T*) (i.e., the slopes of each series of data in Fig. 6), we can obtain the Barkhausen volume *V*, because it is known that T = 300 K and *M*_*s*_ ≈ 0.7 ± 0.1 *μ*_*B*_/atom for Co_30_Pd_70_ alloy^27,28^. The deduced *V* values are discussed later in this paper.

### Polar magnetic domain reversal

**Figure 7:**
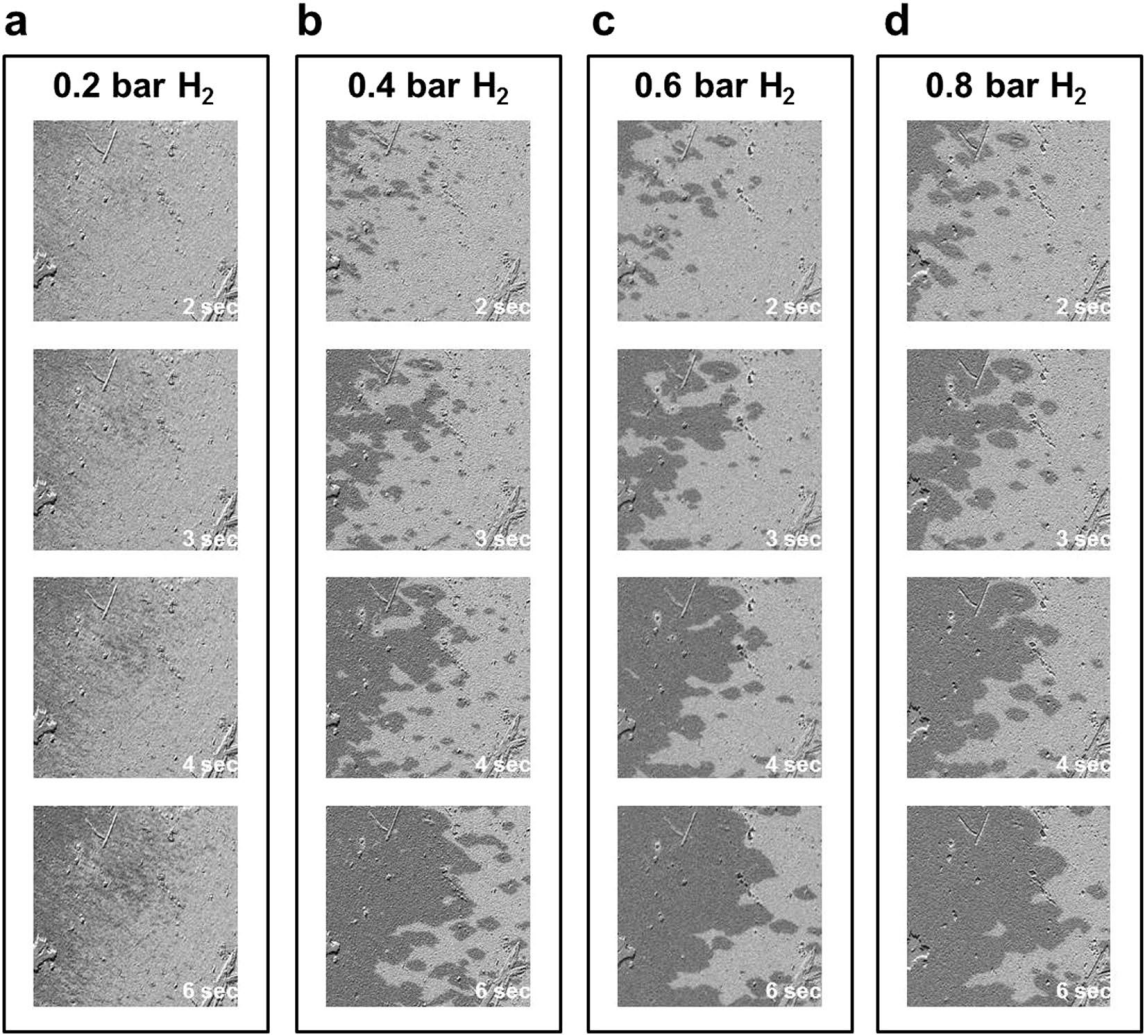
Time-dependent polar Kerr images taken under (**a**) a vacuum, (**b**) 0.2 bar H_2_, (**c**) 0.6 bar H_2_ and (**d**) 0.8 bar H_2_. The four series of Kerr images were taken over the same area, at 2, 3, 4, and 6 s after the application of the reverse magnetic field, which was approximately 0.92 times the magnetic coercivity field in each condition. The image size was 450 × 450 *μ*m^2^.

Because the 25-nm Co_30_Pd_70_ alloy film presented canted magnetization (i.e., the coexistence of longitudinal and polar moment), a characterization of polar magnetization reversal behavior was also performed, similar to the aforementioned analysis of longitudinal domain formation and DW motion. Figure 7 shows the polar MOKE images recorded 2–6 s after the application of a reverse magnetic field: approximately 0.92 × H_*c*_. The polar Kerr images were captured with a symmetric incident light so that the contribution from longitudinal moment was nullified and only the polar moment contributed to the image contrast.

**Figure 8:**
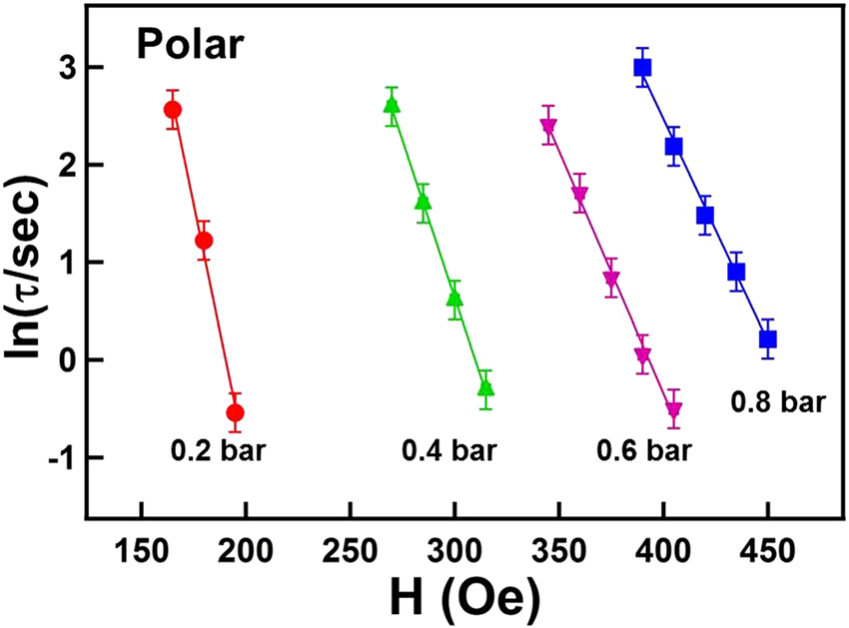
*ln*(*τ*) plotted as a function of the applied reversal magnetic field H. The time constant *τ* under the various magnetic fields and H_2_ pressures were obtained by fitting the polar magnetization reversal curves with Eq. (1). The dots represent the experimental data and the solid lines represent the fitted results obtained using Eq. (4).

Consistent with the results of longitudinal measurement shown in Figs 3 and 4, no observable magnetic domain structure for the Co_30_Pd_70_ alloy film existed under a vacuum condition. The gray scale of the polar Kerr images continuously and monotonically changed from bright to dark without any observable contrasting structures. The reverse magnetic field only triggered magnetization switching of the uniformly spread nucleation sites without observable domain propagation. As exhibited in Fig. 7(a), under 0.2 bar H_2_, many tiny needle-like domains appeared after 2 s and subsequently propagated toward the right-hand side. In Fig. 7(b), the number of reverse nucleations under 0.4 bar H_2_ was considerably fewer than those under 0.2 bar H_2_. DW motion and domain merging also became faster and more evident. The increase in H_2_ gas pressure from 0.4 to 0.8 bar only slightly reduced the number of nucleations; additionally, DW motion was slightly faster than that at 0.4 bar. The time dependence of the polar magnetization reversal process was monitored under various magnetic fields and H_2_ gas pressures. The time constant *τ* values were obtained by fitting the time-dependent magnetization reversal data with Eq. (1). Accordingly, Fig. 8 shows the *ln*(*τ*) − *H* plot with the linear fitting lines. The *ln*(*τ*) values followed the linear equation of Eq. (4), and from the slope of the fitting lines, the corresponding Barkhausen volume *V* can be deduced using the parameters of T = 300 K and *M* ≈ 0.7 ± 0.1 *μ*_*B*_/atom.

**Figure 9:**
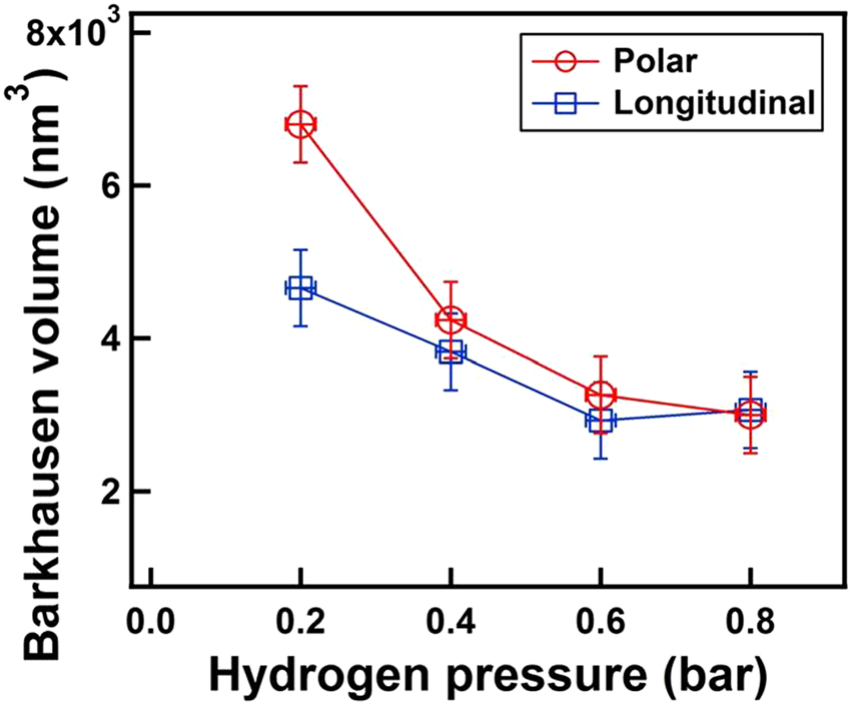
Barkhausen volume deduced from the fitting in Figs 6 and 8 for the longitudinal (open square) and polar (open circle) magnetization reversal, respectively.

Figure 9 summarizes the Barkhausen volume *V* deduced from the fitting in Figs 6 and 8 for the longitudinal and polar magnetization reversal, respectively. Because a 25-nm Co_30_Pd_70_ alloy film was used in this study, we can obtain the activation area in each activation event of magnetization reversal by dividing *V* with 25 nm. In the polar Kerr measurement, whenH_2_ pressure was increased from 0.2 to 0.8 bar, *V* monotonically decreased from 6800 ± 500 to 3000 ± 500 nm^3^; correspondingly, the activation area *A* decreased from 272 ± 20 to 120 ± 20 nm^2^. In the longitudinal Kerr measurement, *V* decreased from 4600 ± 500 to 3000 ± 500 nm^3^ when the H_2_ pressure increased from 0.2 to 0.8 bar; correspondingly, the activation area *A* decreased from 184 ± 20 to 120 ± 20 nm^2^. The deviation between polar and longitudinal measurements may be due to the variation of moment canting angle. In addition, the distribution of *E*_*A*_ over the sample could also affect the analysis result, as proposed by Bruno *et al*. Nevertheless, despite the unavoidable limited error, the polar and longitudinal measurements exhibit a consistent and convincing tendency for *V* to monotonically decrease with H_2_ gas pressure. From Eq. (3), the activation energy in a single magnetization reversal event was proportional to *V*. Therefore, the hydrogen-induced monotonic decrease of *V* suggested that the presence of hydrogen content in Co_30_Pd_70_ alloy films reduced the activation energy of magnetization reversal events and thus promoted DW motion^25,26^. On the other hand, when the H_2_ gas in the sample environment was limited, large amounts of activation energy were required for a single magnetization reversal event. This finding indicated that activating a single event became harder at room temperature and thus the DW motion was almost unobservable. Accordingly, instead of DW motion, nucleation dominated magnetization reversal when the alloy film was under a vacuum or limited H_2_ pressure (<0.2 bar).

### Domain wall velocity

**Figure 10:**
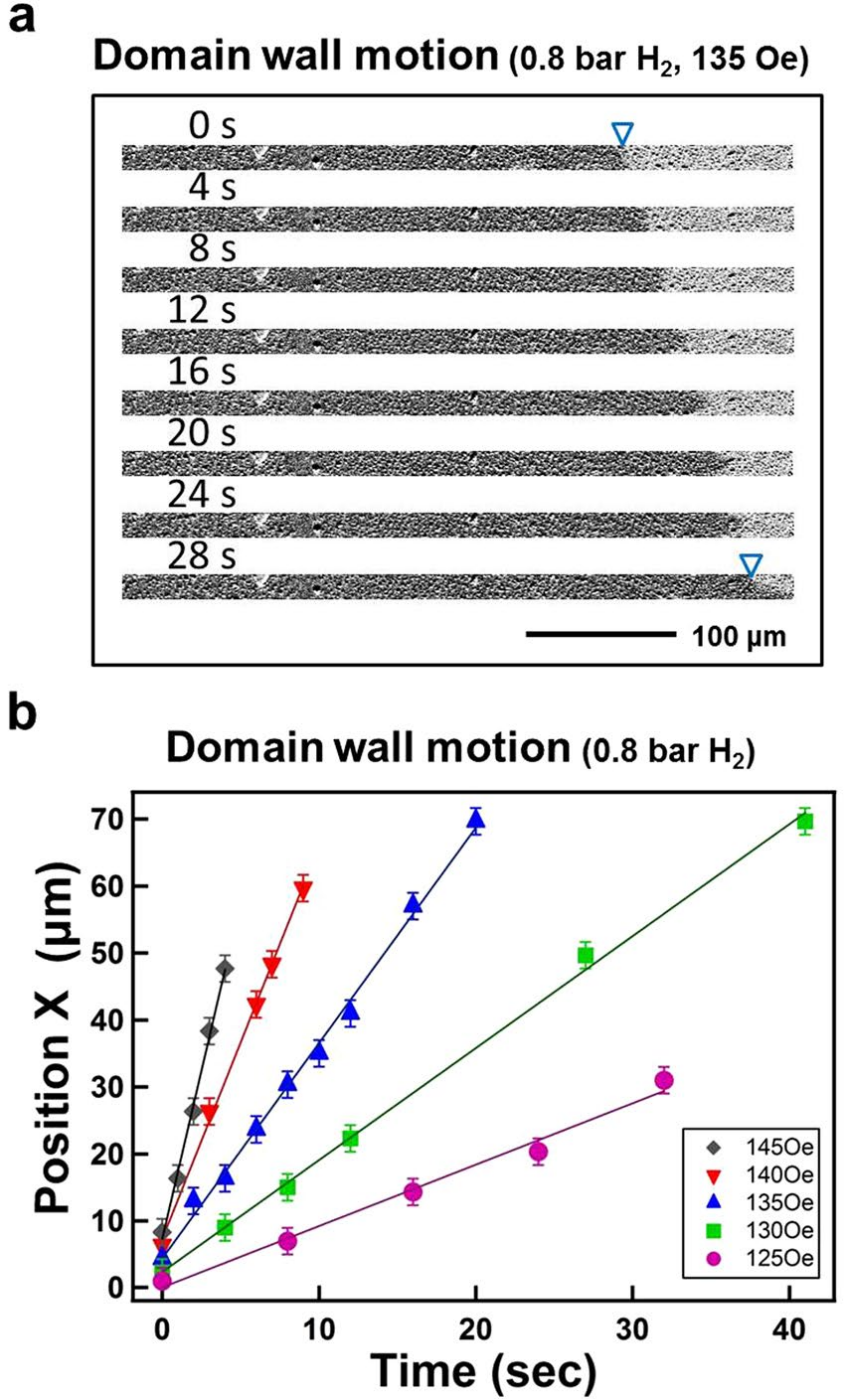
Time-dependent polar Kerr images for the monitoring of DW motion. The triangles indicated the initial and final position of the DW. The Kerr images were recorded under 0.8 bar H_2_ with a reversal magnetic field of 135 Oe. (**b**) Summarized DW position X plotted as a function of time. A series of measurements were taken under 0.8 bar H_2_ with various reverse magnetic fields. Solid notations represent the experimental data; the lines were the linear fitting to the experimental data.

**Figure 11:**
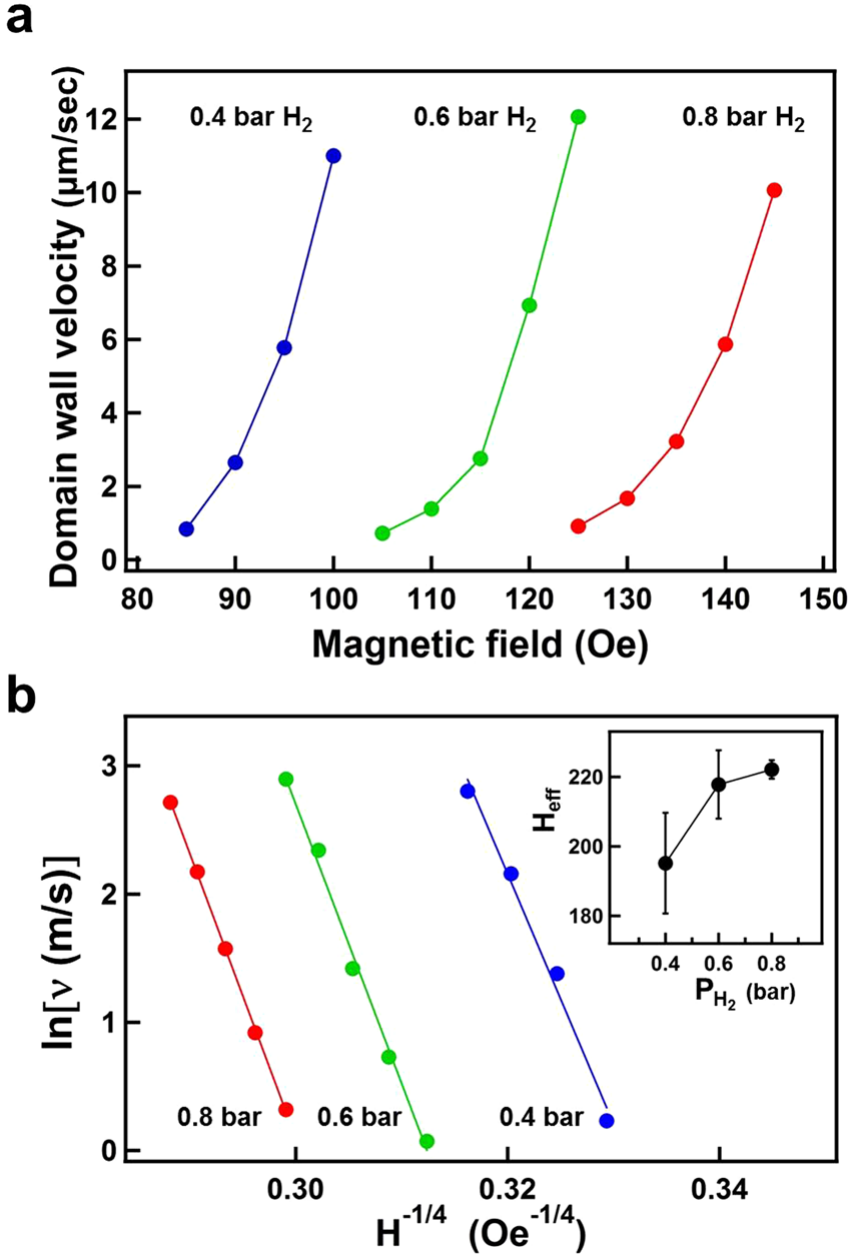
(**a**) DW velocity values under various H_2_ gas pressures were plotted as a function of the magnitude of the reversal magnetic field. (**b**) ln(*υ*) plotted as a function of *H*^−1/4^. The lines are linear fitting to the experiment data (solid notations) obtained using Eq. (5). Inset is the fitted slopes *H*_*eff*_ = (*U*_*c*_/*k*_*B*_*T*)^4^*H*_*crit*_, plotted as a function of the H_2_ gas pressure of the measurement condition.

Figure 10(a) shows the time-dependent polar Kerr images for the monitoring of polar DW motion. The triangles indicate the initial and final position of the DW. The Kerr images were recorded under 0.8 bar H_2_ with a reverse magnetic field of 135 Oe in the polar direction. The invariant defect positions exhibited on the left side of the images in Fig. 10(a) ensured that measurement was always conducted in the same area and that image drift was negligible. In Fig. 10(b), the DW position (*X*) is plotted as a function of time. A series of measurements were taken under 0.8 bar H_2_ with various reverse magnetic fields. The solid notations represent the experimental data, and the solid lines are the linear fitting of the experimental data. With a larger magnetic field, the slope of the *X* − *t* line was larger, indicating a faster DW velocity. As demonstrated in Figs 4 and 7, the magnetic domain reversal under 0.2 bar H_2_ was dominated by numerous nucleation sites and the subsequent DW motion. Under 0.4–0.8 bar H_2_, DW motion dominated the magnetization reversal. Thus, DW motion in the same area of the sample could be measured. Figure 11 summarizes the DW velocity values under various H_2_ gas pressures: 0.4–0.8 bar H_2_, plotted as a function of the magnitude of the magnetic field. The obtained DW velocity is approximately 10^−6^–10^−5^ m/s, indicating that the DW motion was in a thermally activated creep regime^29–32^. At a temperature of 0 K, depinning transition of magnetization only occurred with a sufficiently large driving force (i.e., the external magnetic field was required to be above the critical depinning field H_*crit*_). At a finite temperature, despite the external magnetic field being below H_*crit*_, the depinning process was still activated by thermal energy, and thus a finite velocity of DW motion was still observed. While the external force was small (i.e., the magnetic field $H\ll {H}_{crit}$), the thermally activated DW velocity was low and referred to as “creep”^29–31^. Under such conditions, a one-dimensional DW traveling in a weakly disordered two-dimensional medium can be described as the following equation. The domain wall velocity *υ* is expressed as a function of the applied magnetic field H^33,34^.

5 $$\upsilon ={\upsilon }_{0}\cdot \exp \,[-(\frac{{U}_{c}}{{k}_{B}T}){(\frac{{H}_{crit}}{H})}^{1/4}]$$

where *υ*_0_ is a numerical prefactor; *U*_*c*_ is the scaling energy constant, as the pinning energy barrier height of DW motion; and H_*crit*_ is the critical field, determined by the depinning field. In Fig. 11(b), *ln*(*υ*) was plotted as a function of H^−1/4^ for 0.4, 0.6, and 0.8 bar H_2_. The solid lines are linear fitting to the experiment data (solid notations) by Eq. (5). The inset of Fig. 11(b) shows the fitted slopes *H*_*eff*_ = (*U*_*c*_/*k*_*B*_*T*)^4^*H*_*crit*_, plotted as a function of the H_2_ gas pressure of the measurement condition. The change in H_*eff*_ indicated that the energy barrier was modulated by the H_2_ gas pressure. The uncertainty of the fitted slope (i.e., the error bars shown in the inset), was significantly reduced with H_2_ pressure, which indicated that greater hydrogen content in Co_30_Pd_70_ alloy film can promote DW motion that more accurately mirrors the thermally activated model, as described in Eq. (5).

## Discussion

**Figure 12:**
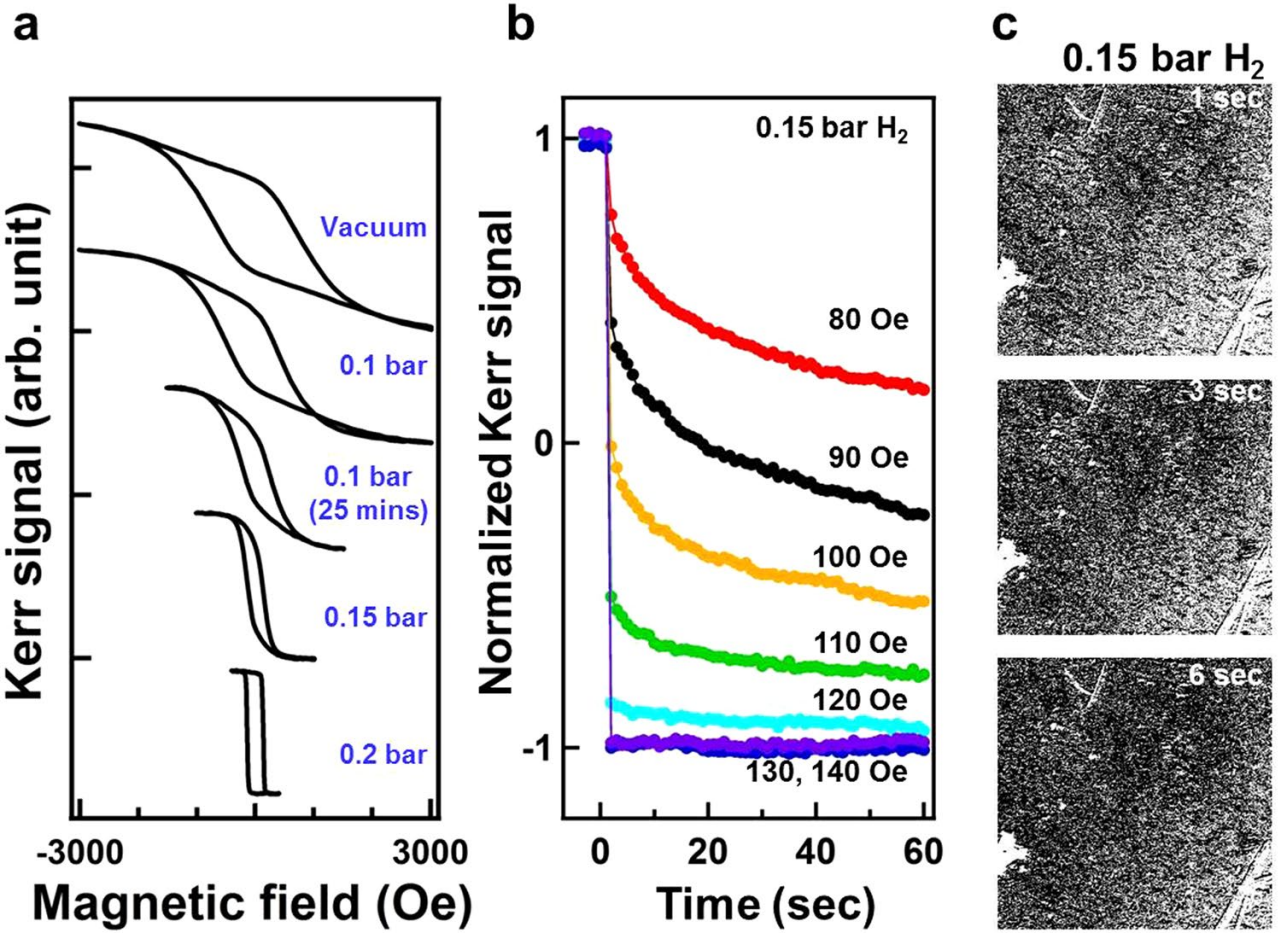
(**a**) Longitudinal MOKE hysteresis loops measured under a vacuum and 0.1–0.2 bar H_2_. (**b**) Time-dependent magnetization reversal curves under 0.15 bar H_2_ with different applied magnetic fields. (**c**) MOKE images recorded at 1–6 s after a reverse magnetic field of 100 Oe was applied under 0.15 bar H_2_. The image size was 450 × 450 *μ*m^2^.

In Fig. 2, the drastic reduction of H_*c*_ from a vacuum to 0.2 bar H_2_ was shown to be correlated to the transition of tiny-nucleation dominance to the appearance of needle-shape domains with DW motion, as revealed in the domain observations in Figs 3, 4 and 7. Why this drastic transition occurred and why the hydrogen content in Co_30_Pd_70_ alloy film triggerred domain formation and promoted DW motion remain unclear. Figure 12 summarizes the observations in the transition region (i.e., under 0.1–0.15 bar H_2_), including (a) MOKE hysteresis loops, (b) time-dependent magnetization reversal curves, and (c) time-dependent magnetic domain images. The MOKE hysteresis loops showed a continual transition of characteristics, such as H_*c*_ reduction and squareness enhancement, in-between a vacuum condition and 0.2 bar H_2_.

The magnetization reversal curves of 0.15 bar H_2_ were composed of a drop immediately after field application and then followed an exponentially behaved curve. These two-step reversal curves indicated the coexistence of swiftly formed tiny nucleations and DW motion. When the applied magnetic field was markedly less than H_*c*_, the immediate drop was small (i.e., few reversal nucleations), and the following exponential curve slowly evolved with a large time constant. When the applied magnetic field was close to H_*c*_, the immediate drop was clear (i.e., many reversal nucleations triggered); however, the following exponential curve still decayed slowly. The time-dependent evolution of magnetic domain under 0.15 bar H_2_ exhibited the spreading of scattered domain, which were composed of tiny nucleations, in contrast to the uniform contrast evolution and invisible nucleations in a vacuum condition (Figs 3(a) and 4(a)). An increase in hydrogen content in the Co_30_Pd_70_ fostered the expansion of tiny nucleations, formed larger magnetic domains, and even promoted DW motion, leading to quick magnetic domain reversal with a small H_*c*_. The mechanism could have been spin-polarized H atoms in the interstitial space that strengthened the magnetic coupling between adjacent Co_30_Pd_70_ grains and thus mediated the DW motion across the grain boundary. As reported by theoretical and experimental studies, not only Co but also Pd were spin-polarized in Co–Pd alloys. Guevara *et al*. revealed that in a Co_30_Pd_70_ alloy, the average magnetic moment of Co and Pd were approximately 2.0 and 0.2 *μ*_*B*_, respectively^27^. Because a hydrogen atom contains only a proton and an electron, its atomic radius is small (approximately 0.52 Å in the ground state); consequently, hydrogen atoms can easily diffuse into materials. From the *X*-ray absorption spectra measurement, Liang *et al*. reported that the hydrogen effect on CoPd alloy can be viewed as an electronic interaction between Co and Pd, wherein the Co acts as an electron donor and Pd acts as an electron receiver^20,35,36^. The charge transfer effect is spin-dependent, due to the fact that the electron band structure of Co is spin-split. The hydrogen absorption/desorption induced charge transfer which fills/withdraws electrons into/from the minority band of Co. Although it was not able to detect the *X*-ray absorption or photoemission spectra of hydrogen atoms, the charge transfer in H-Co and H-Pd bonding was expected. For example, den Broeder *et al*. discovered that the migration of *γ*-phase hydride on the side of a negative electrode was much faster than on the positive side^4^. Their results implied that hydrogen in YH_*x*_ hydride behaved as a negatively charged impurity. Thus, for the hydrogen absorbed in Co_30_Pd_70_ alloy, its pristine unpaired single electron with possible charge transfer from Pd and Co was expected to carry a net spin moment with long-range ordering because of the local field from Co_30_Pd_70_ magnetization. Spin-polarized hydrogen content could mediate the intergrain coupling and thus reduce the activation energy of the single event in magnetization reversal.

From the analysis of the magnetization reversal curve, we deduced the E_*A*_ values for a single thermally activated event. E_*A*_ decreased with the H_2_ pressure (0.2–0.8 bar) (Fig. 9). In the inset of Fig. 11(b), the H_*eff*_ values are deduced from the DW velocity using Eq. (5). In contrast to the E_*A*_ variation, H_*eff*_ increased with H_2_ pressure. Because *H*_*eff*_ = (*U*_*c*_/*k*_*B*_*T*)^4^*H*_*crit*_, the increase in H_*eff*_ was correlated to an increase in U_*c*_ or H_*crit*_. H_*crit*_ was associated with the magnetic anisotropy energy. From the increased H_*c*_ in MOKE hysteresis loops of 0.2–0.8 bar, we can confirmed the enhanced magnetic anisotropy energy with H_2_ pressure. U_*c*_, the pinning energy barrier height of DW motion had similar physical origins as E_*A*_ in magnetization reversal. Thus, U_*c*_ was expected to decrease with H_2_ pressure, similar to E_*A*_, and the increase in H_*eff*_ was was attributed to the enhancement of H_*crit*_ (i.e. the magnetic anisotropy energy). In other words, at 0.2–0.8 bar H_2_, the larger the H_2_ pressure was, the lower the activation energy barrier (E_*A*_ or U_*c*_) for magnetization reversal or DW motion was, whereas the magnetic anisotropy energy as well as the H_*eff*_ increased. The reduction in the activation energy barrier was indicated by the larger domain size with increased H_2_ pressure. Enhanced magnetic anisotropy was revealed by the increase in H_*c*_ during 0.2–0.8 bar H_2_.

## Conclusion

In this study, the microscopic origin of the hydrogen effect on 25 nm Co_30_Pd_70_ alloy thin films was explored through characterization of the time-dependent magnetic domain evolution. From the measurement of Kerr hysteresis loops, we observed significant hydrogen-induced reduction of magnetic coercivity by a factor of 1/5 in a longitudinal direction and enhancement of magnetic remanence from 60% to 100%. Hydrogen inducing considerable modulation of macroscopic magnetic behavior motivated the exploration of its microscopic physical origins. Thus, the microscopic magnetic domain was characterized using a MOKE microscope. Under a vacuum condition, no domain structure on the Co_30_Pd_70_ film was observed. The gray scale continuously changed with the increase in magnetic field because the reversal nucleations were uniformly distributed and the size was below the resolution of the MOKE microscope (approximately 1 *μ*m). Under 0.2 bar H_2_, numerous dot- and needle-shaped domains started to appear. When H_2_ pressure was above 0.4 bar, a clear domain contrast and DW motion were observed. These observations indicated that the magnetic reversal behavior of the Co_30_Pd_70_ alloy film gradually transformed from nucleation dominance to DW-motion dominance when the H_2_ pressure was increased from 1 × 10^−5^ mbar to 0.8 bar.

From quantitative analysis of the time-dependent magnetization reversal curves, the activation energy E_*A*_ decreased with the H_2_ pressure. This analysis revealed that the energy barrier domain reversal was reduced by hydrogen absorption. The DW velocity was approximately 10^−6^–10^−5^ m/s and was obtained by monitoring DW motion in the same area. This slow velocity was in the creep region, which was activated by thermal energy. From comparative experiments with various H_2_ pressures and magnetic fields, more hydrogen content in the Co_30_Pd_70_ alloy film was noted to promote DW motion closer to the behavior of a thermally activated model, which has been widely utilized to describe the experimental results of 2D films and 1D wires.

In summary, the presence of hydrogen atoms in the grain boundary and atomic interstitial space drastically reduced H_*c*_ by mediating domain formation and DW motion. The hydrogen effects on magnetic behavior were observed to be reversible and to have possible future applications in hydrogen sensing and spintronic devices.

## Methods

### Sample preparation

Co_30_Pd_70_ alloy thin films were deposited on SiO_2_/Si(001) substrates by using the coevaporation method with two e-beamheated evaporators in an ultrahigh-vacuum chamber with a base pressure of 3 × 10^−9^ mbar^6,23,24^. The film thickness and alloy composition were determined by the individual calibration of the Co and Pd deposition rate.

### Magnetic measurements

The magnetic properties of the CoPd films for both polar and longitudinal geometries were investigated at room temperature by using a MOKE microscope with a spatial resolution of approximately 1 *μ*m. The MOKE microscope measurement was conducted with a sample holder, which was either sealed in a vacuum with a base pressure of 1 × 10^−5^ mbar or filled with H_2_ gas at various pressures. Accordingly, the time-dependent magnetic domain structure of the Co_30_Pd_70_ alloy films was investigated under various magnetic field strengths in either polar or longitudinal direction and various H_2_ pressures.
